# NOX2-Derived Reactive Oxygen Species in Cancer

**DOI:** 10.1155/2020/7095902

**Published:** 2020-11-27

**Authors:** Hanna Grauers Wiktorin, Ebru Aydin, Kristoffer Hellstrand, Anna Martner

**Affiliations:** ^1^TIMM Laboratory, Salgrenska Center for Cancer Research, Department of Infectious Diseases, Institute of Biomedicin, Sahlgrenska Academy, University of Gothenburg, Sweden; ^2^Molecular Genetics, German Cancer Research Center (DKFZ), Heidelberg, Germany

## Abstract

The formation of reactive oxygen species (ROS) by the myeloid cell NADPH oxidase NOX2 is critical for the destruction of engulfed microorganisms. However, recent studies imply that ROS, formed by NOX2^+^ myeloid cells in the malignant microenvironment, exert multiple actions of relevance to the growth and spread of neoplastic cells. By generating ROS, tumor-infiltrating myeloid cells and NOX2^+^ leukemic myeloid cells may thus (i) compromise the function and viability of adjacent cytotoxic lymphocytes, including natural killer (NK) cells and T cells, (ii) oxidize DNA to trigger cancer-promoting somatic mutations, and (iii) affect the redox balance in cancer cells to control their proliferation and survival. Here, we discuss the impact of NOX2-derived ROS for tumorigenesis, tumor progression, regulation of antitumor immunity, and metastasis. We propose that NOX2 may be a targetable immune checkpoint in cancer.

## 1. Introduction

### 1.1. Distribution and Function of NOX Enzymes

The NOX family of enzymes comprises seven structurally conserved isoforms, *i.e.*, NOX1-5 and DUOX1-2. The only known function of these transmembrane multicomponent enzymes is to catalyze the reduction of molecular oxygen to generate superoxide (O_2_^−^) or hydrogen peroxide (H_2_O_2_) [1, 2]. Superoxide is spontaneously or enzymatically converted to H_2_O_2_ that may be further converted to additional reactive oxygen species (ROS), including myeloperoxidase- (MPO-) derived hypochlorous acid and tyrosyl radical [3].

NOX enzymes differ in distribution between cell types in their subcellular localization and composition of subunits. NOX1 is mainly expressed in the colon, NOX2 on the lysosomal and plasma membranes of myeloid cells where it contributes to phagocyte killing of microbes, NOX3 in the inner ear and fetal tissues, NOX4 in the kidney, NOX5 in lymphoid tissue and testis, and DUOX1-2 in thyroid and gastrointestinal tissues [4, 5]. Low expression levels of NOX1 and NOX4 are also detected in myeloid cells [4, 6, 7], and NOX2 is minimally expressed by hematopoietic stem cells [8]. NOX2 is further expressed at low levels by B cells that may take up and, similar to myeloid cells, degrade microbial pathogens by generating NOX2-derived ROS [9]. Additionally, within dendritic cell (DC) phagolysosomes, NOX2 generates ROS in a process that consumes protons leading to alkalization of this compartment. This protects engulfed peptides from complete degradation by lysosomal proteases, which facilitates their presentation to cytotoxic T cells [10–12].

### 1.2. NOX Enzymes in Cancer

ROS formed from NOX enzymes have been implicated in carcinogenesis [13]. In addition, several NOX enzymes are expressed in malignant tissue and may contribute not only to cancer progression and spread but also to apoptosis of malignant cells. NOX1 is implicated in colon cancer where its ROS-producing activity may enhance tumor cell proliferation and metastasis [14, 15]. Myeloid leukemic cells express high levels of NOX2 that compromises destruction of malignant cells by triggering ROS-induced apoptosis of adjacent antileukemic lymphocytes [16–19]. Stem cell expression of NOX2 has been implicated in leukemogenesis by maintaining survival of leukemic stem cells [8]. NOX2 is further expressed by EBV-infected gastric cancer cells to promote tumor progression [20] and by non-small-cell lung cancer cell lines, where it mediates tumor cell apoptosis [21]. NOX4 is overexpressed in several forms of cancer, including breast cancer, where it may enhance tumorigenesis [22], and prostate cancer, where it promotes apoptosis [23].  Table 1 summarizes the proposed physiological and pathophysiological functions of NOX enzymes.

Additionally, ROS from all cellular sources, including NOX-derived ROS, participate in redox signaling by oxidizing thiol groups on proteins, thus modifying cellular functions and activation status. For example, ROS may oxidize protein tyrosine phosphatases (PTPs) and protein kinase C (PKC) with ensuing effects on differentiation, proliferation, and survival of malignant cells [69–73].

### 1.3. The Myeloid NADPH Oxidase: NOX2

**Figure 1:**
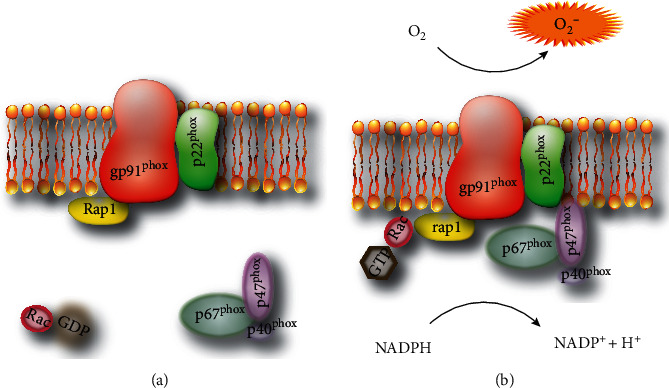
NOX2 in its resting and activated states. In its resting state (a), the membrane-bound and cytosolic subunits of NOX2 are spatially separated. Upon activation (b), the cytosolic subunits assemble with the membrane-bound subunits to generate O_2_^−^.

The first discovered and by far most extensively studied member of the NOX enzyme family, NOX2, is densely expressed by myeloid cells such as monocytes, macrophages, and granulocytes [2]. NOX2 is a complex of membrane-bound and cytosolic subunits that are spatially separated in resting cells. The membrane-bound subunits, gp91^phox^ (also referred to as CYBB or NOX2) and p22^phox^ (CYBA), constitute the catalytic core of the oxidase. The subunits p47^phox^ (NCF1), p67^phox^ (NCF2), and p40^phox^ (NCF4) remain in the cytosol as a complex. Activation of NOX2 may be induced by pathogen-associated molecular patterns, danger-associated molecular patterns, bacterial peptides, growth factors, and cytokines, which trigger the cytosolic subunits p47^phox^ (NCF1), p67^phox^ (NCF2), and p40^phox^ (NCF4) to translocate and assemble at the membrane [5, 74]. Two GTPases, Rac and Rap, are also critical for NOX2 activation [75, 76]. In its GTP-bound form, the cytosolic Rac interacts with p67^phox^ and translocates to the membrane. Rap1 is a membrane protein with a partly unknown function that is required for optimal activation of NOX2 components [77] (Figure 1).

Phagocytes are stimulated to generate NOX2-derived ROS upon encountering microbes in a process referred to as a “respiratory burst.” When the components of NOX2 assemble at the phagolysosome membrane, NOX2 generates intracellular ROS, while assembly at the plasma membrane leads to the formation of extracellular ROS [5, 78]. The respiratory burst is critical for phagocyte-mediated killing of microorganisms as highlighted by the susceptibility to bacterial and fungal infections in patients with chronic granulomatous disease, a rare genetic disorder caused by dysfunction of NOX2 [79–81], and by studies in mice that are genetically deprived of NOX2 [82]. NOX2 deficiency is also associated with hyperactive lymphocytes and autoimmunity in mice and humans, indicating that NOX2-derived ROS also participate in controlling lymphocyte reactivity [83–85]. Additionally, monocyte-derived DCs express NOX2, and the formation of NOX2-derived ROS by pathogen-activated DCs is proposed to reduce the potential transmission of pathogens to secondary lymphoid organs [86].

## 2. Redox Homeostasis

In addition to NOX-mediated formation of ROS, all cells generate ROS during mitochondrial ATP generation. In the process of oxidative phosphorylation, electrons pass through the electron transport chain where the final electron acceptor is oxygen, most of which is converted to water. Superoxide is produced as a byproduct in this process due to incomplete reduction of oxygen to water or premature electron leakage to oxygen [87, 88]. Intracellular levels of ROS affect cellular redox signaling and homeostasis, while ROS released into the surrounding, in particular H_2_O_2_ that is relatively stable and readily crosses cell membranes, may also affect adjacent cells [19, 89–91]. Under resting conditions, when there is a balance between ROS and antioxidants, redox signaling is reversible and regulates physiological processes due to the ability of ROS to reversibly oxidize cysteine residues to thus alter protein function [92, 93]. During environmental stress, infection, and inflammation, including cancer-related inflammation, the cell and tissue concentrations of ROS may increase beyond the capacity of the antioxidant defense systems. Such “oxidative stress” may result in irreversible oxidation and damage to proteins, lipids, and DNA [92]. Details regarding redox homeostasis and its impact on cancer have recently and comprehensively been reviewed [94, 95] and is beyond the major scope of this overview.

To avoid ROS-inflicted cell damage, several cellular systems that neutralize ROS are induced in an oxidative environment. The transcription factor Nrf2 is a key regulator of production of antioxidative enzymes within cells. In resting conditions, Nrf2 is bound to Keap1 in the cytoplasm, which prohibits Nrf2 from inducing gene transcription. Upon oxidation of cysteine residues in Keap1, Nrf2 is released and translocates to the nucleus where it binds to antioxidant response elements [96]. This process stimulates the transcription of Nrf2 target genes with cytoprotective functions. These include NAD(P)H quinone oxidoreductase 1, which catalyzes the reduction of reactive quinones that otherwise cause oxidative stress [97], heme oxygenase-1 (HO-1) that catalyzes the breakdown of heme [98], glutamate-cysteine ligase catalytic and modifier that catalyzes the rate-limiting step in synthesis of the endogenous antioxidant glutathione (GSH) [99], and thioredoxin reductase 1 that reduces peroxiredoxins of relevance to the detoxification of reactive peroxides, including H_2_O_2_ and peroxynitrite [100].

**Figure 2:**
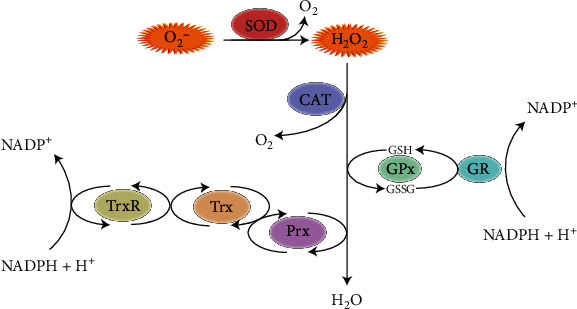
Mediators of redox homeostasis during the metabolism of O_2_^−^. Superoxide dismutase (SOD) catalyzes the conversion of O_2_^−^ to H_2_O_2_. Catalase (CAT) metabolizes H_2_O_2_ into O_2_ and H_2_O. Glutathione peroxidase (GPx) detoxifies H_2_O_2_ by oxidation of reduced glutathione (GSH) to its oxidized form, GSSG. Intracellular GSH levels are regulated by glutathione reductase (GR). H_2_O_2_ is also metabolized by peroxiredoxin (Prx) that is recharged by thioredoxin (Trx). Trx is kept in a reduced state by thioredoxin reductase (TrxR).

Other cellular antioxidant enzymes include superoxide dismutase, catalase, glutathione peroxidase-1, peroxiredoxins, and thioredoxin. Together with the nonenzymatic antioxidant GSH, these antioxidant enzymes are assumed to provide the most efficient protection from oxidative damage (Figure 2). Additional nonenzymatic scavengers of ROS include naturally occurring metabolites, vitamins (such as vitamins C and E) and iron chelators that prevent formation of hydroxyl radicals in the Fenton reaction [101, 102].

## 3. ROS and Cancer

### 3.1. Cancer-Related Oxidative Stress

Cancer may be associated with oxidative stress, *i.e*., an imbalance between the production and detoxification of ROS. Rapidly proliferating cancer cells have a high energy demand and therefore exhibit enhanced cellular respiration. Consequently, cancer cells generate enhanced levels of mitochondrial-derived ROS [89]. Growth factors and integrins, which are often produced at enhanced levels in cancer tissues, also contribute to enhanced NOX-derived ROS production [103] and, as reviewed above, several cancer histiotypes exhibit dysregulated expression of NOX enzymes [8, 14–17, 20–23]. Furthermore, solid and metastatic tumors are often infiltrated by NOX2^+^ myeloid cells that may release ROS leading to an oxidized tumor microenvironment [104–108]. The extracellularly released ROS from myeloid cells affect redox regulation in adjacent tumor cells and may inactivate T cells and NK cells, thus compromising immune-mediated killing of malignant cells [19, 90, 91, 109–111]. Hypoxia is a common feature of the microenvironment of tumors that activates the hypoxia-inducible factor (HIF) family of transcription factors. HIFs mediate cellular adaptation to low oxygen levels and may influence several aspects of cancer such as promoting neovascularization [112], increasing cell survival [113], stimulating metastasis [114, 115], and conferring resistance to chemotherapeutics [116]. ROS may induce the activation of HIF-1*α*, a member of the HIF family of transcription factors, and thereby stimulate HIF-related cancer events [117].

The arguably most established role of ROS in cancer is its capacity to damage DNA with ensuing mutations and risk of cancer initiation and progression. Typically, deoxyguanosine is oxidized to 8-oxo-2-deoxyguanosine that may pair with adenine instead of cytosine, which promotes mutations in oxidatively stressed cells [118–121]. Overexpression of NOX enzymes, including NOX4, DUOX1, and DUOX2, has been shown to generate excessive H_2_O_2_ that may cause local tissue injury and DNA damage, thus resulting in the formation of a premalignant niche. NOX-derived ROS may thus contribute to tumor initiation and to tumor progression by inducing further DNA damage [122, 123].

Moreover, many cancer-related events, such as cell cycle proliferation, invasion, epithelial-to-mesenchymal transition, and metastasis are subject to redox regulation [47, 69–71, 73, 124–130]. For example, growth factors such as PDGF and EGF stimulate the PI3-K-AKT and RAS-MEK-ERK pathways, which are key regulators of cell proliferation and survival [131, 132]. These growth factors also stimulate NOX enzymes to produce ROS. The kinases in the PI3-K and RAS pathways phosphorylate target proteins, while PTPs serve to remove phosphate groups from proteins. This phosphorylation/dephosphorylation circuit alters protein function and controls cellular functions [133–135]. ROS may oxidize thiol groups in PTPs resulting in their inactivation. As a consequence, signaling along these pathways is boosted in an oxidative environment where PTPs are inactivated, and cancer cells may thus respond more vigorously to stimulation by growth factors [134, 135].

An additional example of the effects of ROS on PTPs is the inactivation of PTPs in pancreatic cancer cells that results in sustained activation of Janus kinase 2, which in turn activates signal transducer and activator of transcription (STAT) and antiapoptotic proteins to enhance tumor cell survival [72]. ROS may also oxidize and thus activate PKC; thereby, ROS modulate several PKC-dependent activities within cells [126, 136]. ROS have been proposed to enhance the tissue-invasive properties of cancer cells by modulating the function of mitogen-activated protein kinases via oxidation of PTPs and PKC [124–126]. However, as overproduction of ROS by cancer cells may trigger their apoptosis, the clinical efficacy of many therapies relies on induced ROS production in cancer cells, as further discussed below.

Tumor cells often show enhanced levels of antioxidative enzymes, presumably to resist the toxicity from the generation of NOX- and mitochondria-derived ROS [89]. In addition, tumor cells may acquire mutations that further boost antioxidative responses, thereby contributing to tumor cell resistance to oxidative stress. Approximately 30% of human lung cancers thus carry mutations in either Keap1 or Nrf2, resulting in Nrf2 stabilization and enhanced production of endogenous antioxidants [137]. One of the antioxidants controlled by Nrf2 is HO-1 that reduces intracellular levels of free heme; this, in turn, stabilizes the transcription factor BACH1 to activate transcription of genes that promote glucose uptake, glycolysis, and lactate secretion in the Warburg reaction [138]. Accordingly, BACH1 activation was shown to stimulate glycolysis-dependent metastasis of lung cancer cells [137, 138]. Thus, an antioxidative response by tumor cells, or antioxidative treatment strategies such as scavengers of ROS, may enhance tumorigenesis and metastasis by modulating tumor metabolism in favour of glycolysis.

### 3.2. Targeting NOX2 in Experimental Cancer Models

The development of knockout mice with NOX2 deficiency has been instrumental in studies on the role of ROS in cancer from sources other than mitochondria. Mice with deficiency in the NOX2 subunit Ncf1 show reduced growth or incidence of melanomas and the Lewis lung carcinoma tumors, whereas the growth of spontaneously arising prostate carcinoma or methylcholanthrene-induced sarcoma is not affected [38, 139].

Studies in knockout mice imply a role for NOX2 in metastasis. Mice deficient in the NOX2 subunit Cybb thus show reduced lung metastasis after intravenous inoculation of melanoma cells and a lower incidence of spontaneously formed metastases from surgically removed melanomas [37, 140, 141]. The targeting of NOX2 by systemic treatment with the NOX2 transduction inhibitor histamine dihydrochloride (HDC) reduced the formation of lung melanoma metastases in wild-type but not in *Nox2*-deficient mice. Effects of NOX2 repression on hematogenous metastasis were absent after the depletion of NK cells *in vivo* and absent also in interferon-*γ*- (IFN-*γ*-) deficient mice. These results thus imply that NOX2-derived ROS trigger the formation of melanoma metastasis by downmodulating NK cell functions, and that genetic or pharmacological inhibition of NOX2 restores tumor cell clearance exerted by IFN-*γ*^+^ NK cells [37]. These results were confirmed and extended by Van der Weyden et al. showing that hematogenous metastasis was markedly reduced in mice genetically depleted of any of the major NOX2 subunits (Cyba, Cybb, Ncf1, Ncf2, and Ncf4) and that tumor tissues of NOX2-deficient mice showed a marked increase of antineoplastic lymphocytes [141]. In accordance with the latter finding, treatment with the NOX2 inhibitor HDC resulted in enhanced NK cell counts in the lungs of wild-type mice with pulmonary melanoma metastases, but not in corresponding lungs from *Nox2*-deficient mice [37].

HDC suppresses ROS formation by exerting agonist activity at histamine type 2 receptors (H_2_Rs) [18] and thus inhibits NOX2 signal transduction rather than directly inhibiting, *e.g*., oxidase function or assembly. The detailed mechanisms of NOX2 inhibition and the ensuing protection of antineoplastic lymphocytes are incompletely understood. Myeloid cells deficient of MPO still exerted immunosuppression towards NK cells, which was reversible by HDC-treatment, thus suggesting that O_2_^−^ and H_2_O_2_ are more likely mediators of NOX2-induced immunosuppression than MPO-derived ROS such as, *e.g*., hypochlorous acid or tyrosyl radicals [142]. Additionally, circumstantial evidence links the NOX2-inhibitory properties of HDC to the PI3-K pathway. Activation of PI3-K thus activates Akt and PKC that triggers the assembly and ROS formation of NOX2 [143]. HDC suppresses NOX2-mediated ROS formation induced by fMLF and other bacterial peptides, but does not affect PMA-induced respiratory burst [144]. As fMLF activates the PI3-K pathway [145] whereas PMA directly induces the activation of PKC, these finding thus suggest that HDC, by activating H_2_Rs, targets the PI3-K pathway upstream of PKC in myeloid cells. In support for this hypothesis, PI3-K inhibitors share the NOX2 inhibition exerted by HDC and equally efficiently protect antineoplastic lymphocytes from apoptosis and dysfunction induced by adjacent, ROS-producing myeloid cells [146].

Systemic treatment with HDC *in vivo* suppresses tumor growth in several models of experimental cancer [147]. While these antitumor effects of HDC are likely pleiotropic, it is noteworthy that beneficial effects of treatment with HDC in murine melanoma, lymphoma, and mammary cancer were only observed in NOX2-sufficient mice [32, 35, 37, 148] and that HDC only inhibited growth of NOX2^+^ and not NOX2^−^ leukemic cells in a xenograft setting [35]. Additionally, the efficacy of HDC in reducing murine tumor growth and metastasis relied on the presence of NOX2-expressing Gr1^+^ myeloid cells since the effect was lost upon Gr1^+^ cell depletion [37, 148]. Furthermore, experiments using single-cell suspensions from tumors, spleens, and lungs suggested that ROS formation was confined to the Gr1^+^ cell fraction [37, 148]. These findings, along with results showing that HDC does not reduce metastasis after the depletion of NK cells, support the hypothesis that HDC provides a less immunosuppressive malignant microenvironment that favors NK cell-mediated clearance of tumor cells [37, 83]. Additionally, treatment with HDC was shown to increase the number of tumor-infiltrating effector CD8^+^ T cells in murine lymphoma and to improve the antitumor efficacy of immune checkpoint inhibitors (anti-PD-1 and anti-PD-L1) [148], thus implying that HDC may facilitate also T cell-dependent elimination of tumor cells.

Monocytic leukemic cells recovered from patients with acute myeloid leukemia (AML) frequently express functional NOX2, and studies in xenografted mice support that NOX2 is relevant to the survival and expansion of monocytic AML cells [35, 149]. NOX2-derived ROS have been proposed to stimulate the transfer of prosurvival mitochondria from stromal cells to AML cells [149]. Furthermore, NOX2 inhibition by HDC reduced the expansion of xenografted NOX2^+^ but not of NOX2^−^ human AML cells, presumably by hindering S-phase entry of leukemic cells [35]. These results illustrate that the targeting of NOX2 may reduce malignant expansion independently of functional cellular immunity.

In addition, results obtained in a mouse model of Kras-induced myeloid leukemia showed that *Kras*^+^ NOX2-deficient myeloid cells (*Nox2*^−/−^M-*Kras*^G12D^) expanded slower than their NOX2-sufficient counterparts. In this model, treatment of mice with *N*-methyl-histamine (an H_2_R-selective analogue of HDC that shares the NOX2-inhibitory properties of HDC) reduced leukemic expansion and prolonged the survival of NOX2-sufficient but not of NOX2-deficient mice. *N*-Methyl-histamine-treated mice harbored leukemic cells with reduced intracellular ROS levels, reduced DNA oxidation, and reduced double-stranded DNA breaks [150]. These results thus imply that NOX2-derived ROS may promote genomic instability and malignant expansion in Kras-induced leukemia. NOX2 may also support myeloid expansion of murine *Bcr-Abl1*^+^ cells as transplantation of NOX2^+^*Bcr-Abl1*^+^ cells into irradiated mice causes a more rapidly expanding and severe leukemia than the transfer of NOX2-deficient *Bcr-Abl1*^+^ cells [8, 151].

## 4. Myeloid-Derived Suppressor Cells and NOX2

### 4.1. Myeloid Cells within the Tumor Microenvironment

The presence of cytotoxic lymphocytes, including CD8^+^ T cells and/or NK cells, in the microenvironment of human cancer tumors is typically prognostically favorable, while the presence of infiltrating myeloid cells often, although not invariably, predicts poor survival [104–107, 152–159]. Hence, a high ratio of tumor-infiltrating T cells to myeloid cells entails favorable prognosis in several cancer forms including lung cancer, bladder cancer, glioblastoma, prostate cancer, and renal cell carcinoma [160–166]. In recent years, the neutrophil to lymphocyte ratio and the monocyte to lymphocyte ratio in peripheral blood have emerged as readily available and independent predictors of poor survival in several forms of solid cancer [167], thus underscoring that myeloid cell-induced immunosuppression may impact adversely on cancer prognosis.

Myeloid-derived suppressor cells (MDSCs) are immature and immunosuppressive myeloid cells that accumulate in the tumor microenvironment and in the periphery in patients with cancer. MDSCs comprise pathologically induced myeloid cells of the monocytic (M-MDSCs) and granulocytic (G-MDSC) linages that suppress T cells and NK cells by several mechanisms, including enhanced production of immunosuppressive NOX2-derived ROS, arginase, nitric oxide (NO), TGF-beta, and IL-10 [168]. MDSCs are thus assumed to favor immune escape in cancer [169, 170]. MDSCs and other myeloid cells are attracted to tumors in response to cytokines such as CCL2 and CSF1 for M-MDSCs and CXCL1 and CXCL8 for G-MDSCs [171]. Once in the tumor microenvironment, M-MDSCs may differentiate into tumor-associated macrophages (TAMs) or DCs. TAMs may also originate from infiltrating monocytes and tissue-resident macrophages [172]. MDSCs and TAMs may release soluble molecules such as cytokines, prostaglandins, chemokines, interleukins, and growth factors into the tumor microenvironment that may contribute to the formation of premetastatic niches, promote angiogenesis, promote tumor cell survival, and enhance tumor cell invasion [173, 174]. These properties of MDSCs and TAMs may, in part, account for the unfavorable association between myeloid cell tumor infiltration and prognosis.

TAMs exhibit either M1 or M2 polarization. The M1-polarized TAMs express iNOS and TNF and are denoted proinflammatory, whereas the M2-polarized TAMs produce the L-arginine-depleting enzyme arginase and secrete IL-10 to compromise immune activation [171, 175]. M1 and M2 macrophages both express NOX2, although the expression level is higher in M1 macrophages [176]. Mice lacking NOX1 and NOX2 showed reduced M2 macrophage polarization, while single knockout of NOX1 or NOX2 did not [6]. Hence, in the Lewis lung carcinoma model, wild-type and NOX1/NOX2 double-knockout mice showed a similar degree of TAM infiltration, while the content of M2-TAMs was reduced in the double-knockout mice along with reduced tumor growth [6]. These results imply that inhibition of NOX enzymes may favor M1 polarization in cancer; however, studies of nonmalignant inflammation (spinal cord inflammation in mice) suggest that inhibition of NOX2 instead reduces M1 polarization [177], and further studies are required to define the impact of NOX enzymes on macrophage polarization.

In contrast to MDSCs and M2-TAMs, the intratumoral accumulation of other myeloid cells, such as DCs and M1-polarized TAMs, may indicate favorable cancer prognosis [178–181]. Tumor-infiltrating DCs initiate the induction of tumor-specific T cell responses and are thus critical to evoke antitumor immunity, and M1 polarized macrophages may contribute in the killing of tumor cells [182]. While the favorable impact of the presence of M1-polarized macrophages in cancer tumors is well established, the subdivision of macrophages into distinct populations is challenged by reports showing that TAMs often display features of both M1 and M2 subsets [183, 184].

### 4.2. Immunosuppression by MDSC-Derived ROS

Early studies showed that MDSCs displayed enhanced expression of NOX2 as a result of the activation of the transcription factor STAT3 [185, 186]. The formation of NOX2-derived ROS is considered a major immunosuppressive action mediated by MDSCs, in particular by G-MDSCs [148, 186, 187], and ROS-producing MDSCs or other immunosuppressive myeloid cells thus induce apoptosis or dysfunction in adjacent lymphocytes such as NK cells and T cells [19, 91, 188–190]. ROS induce activation of ERK1/2 in lymphocytes, which results in PARP-1-dependent accumulation of poly-ADP-ribose (PAR) and parthatanosis (a form of apoptosis) [191].

In addition, MDSC-derived ROS inhibit antigen-specific CD8^+^ T cell responses and may thus selectively eradicate antitumor T cell clones [188]. The immunosuppression exerted by ROS towards T cells has been linked to nitration of the T cell receptor (TCR) and occurs when ROS react with NO to form peroxynitrite during MDSC-T cell interactions. Nitration was proposed to induce a conformational change of the TCR, and T cells thus display reduced affinity for MHC-peptide complexes [192]. This effect was linked to ROS as MDSCs with dysfunctional NOX2 did not suppress antigen-specific T cell responses [186]. On a similar note, MDSCs isolated from mice systemically treated with the NOX2 inhibitor HDC produced lower levels of ROS and were less prone to suppress T cells *ex vivo* [148].

### 4.3. ROS as Inhibitors of Myeloid Cell Differentiation

**Figure 3:**
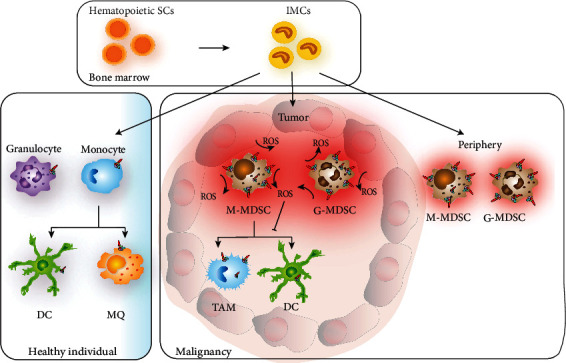
Myeloid cell differentiation in healthy individuals and in cancer patients. Hematopoietic stem cells (SC) differentiate into immature myeloid cells (IMCs) in bone marrow. In healthy individuals, IMCs rapidly differentiate into mature myeloid cell populations in the periphery. In cancer, however, myeloid cell differentiation is often impaired, and the IMCs may be activated to become monocytic or granulocytic myeloid-derived suppressor cells (M- and G-MDSCs, respectively) within tumors and in the periphery. MDSCs show upregulated NOX2 expression and increased production of reactive oxygen species (ROS), in particular in the G-MDSCs. The M-MDSCs may differentiate into tumor-associated macrophages (TAM) or dendritic cells (DC), and the differentiation may be inhibited by excessive intracellular ROS levels.

MDSCs isolated from mice with myeloid cells that cannot generate NOX2-derived ROS, *i.e.*, *Stat3* or *Nox2* knockout mice, are prone to differentiate towards mature macrophages and DCs [186, 193] suggesting that NOX2-derived ROS inhibit myeloid cell maturation and thus promote the accumulation of immature MDSCs. Furthermore, the antioxidant N-acetyl cysteine (NAC) was found to trigger differentiation of MDSCs [194]. Similarly, all-*trans*-retinoic acid (ATRA), which upregulates the antioxidant glutathione synthase and thus reduces intracellular ROS, stimulates the differentiation of MDSCs in murine tumor models and of MDSCs isolated from cancer patients [195–198]. In agreement with these reports, treatment with the NOX2 inhibitor HDC reduces the accumulation of tumor-infiltrating MDSCs in EL-4 thymoma-bearing mice. The reduction of tumor-infiltrating MDSCs was accompanied by augmented levels of intratumoral DCs and by improved maturation of human DCs from monocytes [32, 148].  Figure 3 summarizes aspects of NOX2-mediated regulation of myeloid cell differentiation in cancer.

## 5. Targeting ROS in Human Cancer

While low ROS levels in cells are reportedly mitogenic due to the activation of the PI3-K-AKT and RAS-MEK-ERK pathways [131, 132], high ROS levels are toxic to numerous cell types including cancer cells [92, 118–121]. Several chemotherapies, as well as radiotherapy and photodynamic therapy, trigger excessive ROS production within cells. Oxidants may thus contribute to the elimination of tumor cells and to the toxicity of chemotherapeutics [199]. In addition, several antitumor agents, including erlotinib and silibinin, trigger overproduction of ROS via NOX enzymes, which contributes to killing tumor cells [21, 23].

Despite that increased intracellular ROS levels may induce killing of malignant cells, ROS have also been ascribed protumorigenic properties. Antioxidative strategies have thus been evaluated for human cancer therapy and prevention. Such strategies include ROS scavengers such as NAC, vitamin E, and beta-carotene that are aimed at reducing oxidative stress [200–202]. These studies, as well as animal experiments comprising the administration of ROS scavengers in cancer treatment, have shown partly divergent results. Whereas some studies support that antioxidants reduce the risk of cancer [200–202], other studies, in particular those involving the administration of antioxidants to smokers to prevent lung cancer, imply enhanced cancer risk by the administration of antioxidants[203].

The mechanisms explaining the partly opposing results in studies of broad antioxidants in cancer remain to be elucidated. Recent studies imply that antioxidants trigger the activation of the transcription factor BACH1 that stimulates a metabolic reprogramming of cancer cells in favor of glycolysis, which enhances their capacity to metastasize [137, 138]. These findings may appear counterintuitive in light of the abovereferenced reduction of metastasis induced by HDC and other NOX2 inhibitors that act by reducing ROS levels. However, a noticeable difference between global antioxidants and HDC is that HDC targets NOX2-derived ROS formation only in myeloid cells that coexpress H_2_R and NOX2. HDC or other NOX2-inhibitory strategies are hence unlikely to alter metabolically generated ROS.

ATRA is used in the treatment of acute promyelocytic leukemia where the leukemic cells carry a PML-RARA translocation giving rise to a block in myeloid cell differentiation and development of leukemia. ATRA releases this block and allows the differentiation of immature leukemic promyelocytes into mature granulocytes [204]. ATRA may also promote the differentiation of MDSCs by neutralizing intracellular ROS [195–198]. ATRA exerts antitumoral effects in several murine models [205, 206] and has been investigated in combination with immunotherapies such as IL-2 and DC vaccines in renal cell carcinoma and non-small-cell lung cancer [205–207]. The efficacy of ATRA combined with ipilimumab is currently assessed in stage IV melanoma (ClinicalTrials.gov identifier: NCT02403778).

The NOX2-inhibitor HDC is used in conjunction with low-dose IL-2 within the EU to prevent relapse of AML in the postchemotherapy phase [208]. HDC acts on H_2_Rs expressed on the surface of normal and leukemic myeloid cells to inhibit production of NOX2-derived ROS [208, 209]. *In vitro* studies support that HDC promotes cellular immunity by protecting subsets of cytotoxic lymphocytes against ROS-induced inactivation [19, 91] and that these effects of HDC are markedly enhanced by the coadministration of NK and T cell activators such as IL-2 [111]; however, complementary or alternative mechanisms are conceivable, including HDC-induced differentiation of AML cells [19, 35, 208]. While the side-effects of HDC/IL-2 were typically mild and transient with minimal impact on global health [208, 210], the incidence of grade 1/2 arthralgia and myalgia was slightly but significantly higher in treated patients. It may thus be speculated that HDC/IL-2 induces autoimmunity similar to that observed in NOX2-deficient CGD patients and in experimental animals that are devoid of functional NOX2 [83].

## 6. Conclusion

While details regarding the contribution by NOX2-derived ROS for the induction and progression of cancer remain to be elucidated, it seems likely that the impact of NOX2 is confined mainly to primary and metastatic tumors that are infiltrated by immunosuppressive NOX2^+^ myeloid cells and to myeloid leukemias, where the malignant clone comprises NOX2^+^ cells. In cancer, NOX2 may contribute to the immunosuppression exerted by myeloid cells, in part by producing extracellular ROS that trigger dysfunction in adjacent lymphocytes. Recent studies show that NOX2 promotes tumor growth and metastasis and that intact NOX2 is crucial for self-tolerance, thus fulfilling the criteria of an immune checkpoint [83]. Inhibition of NOX2-derived ROS may thus relieve immunosuppression in cancer and may act in synergy with cancer immunotherapies such NK and T cell-activating cytokines or checkpoint inhibitors.

## Figures and Tables

**Table 1:** Tissue distribution, function, and cancer relevance of NOX enzymes.

Enzyme	Tissue expression (high to low)	Function	Cancer relevance
NOX1	Colon, uterus, prostate [24–28]	Repair of colon mucosa	Colon [14, 15, 29, 30] and prostate [31] cancers
NOX2	Myeloid cells [8, 32–34]	Host defense against pathogens, lymphocyte homeostasis, stem cell maintenance, myeloid cell differentiation	Myeloid leukemia [35, 36], melanoma [37, 38], lymphoma [32]
NOX3	Inner ear, fetal tissue [39–41]	Otoconia synthesis, organogenesis	Hepatocellular carcinoma [42]
NOX4	Kidney [43, 44]	Oxygen sensing^∗^	Renal [45, 46] and ovarian [47] cancers, glioma [48], melanoma [49]
NOX5	Lymphoid tissue, testis [50, 51]	Lymphocyte differentiation, spermatozoa motility	Prostate cancer [52, 53], Barrett's esophageal adenocarcinoma [54]
DUOX1	Thyroid, respiratory tract [55–57]	Hormone synthesis, innate airway host defense	Thyroid [58, 59] and lung cancer[60, 61]
DUOX2	Thyroid, gastrointestinal tract [55, 62–65]	Hormone synthesis, regulation of gut microbiota/mucosa interactions	Thyroid [58, 66] and pancreatic cancer [67, 68]
